# Visioning future transport systems with an integrated robust and generative framework

**DOI:** 10.1038/s41598-023-30818-2

**Published:** 2023-03-15

**Authors:** Peraphan Jittrapirom, Femke Bekius, Karoline Führer

**Affiliations:** 1 Nijmegen School of Management, Radboud University, Nijmegen, The Netherlands; 2 Delft University of Technology, Delft, The Netherlands

**Keywords:** Civil engineering, Energy and society, Sustainability

## Abstract

Visioning has been widely adopted in transport planning as a method to support explorations of possible future transport systems over a long time horizon. There are vast variations in how visioning is applied but given a clear association between visions and the long-time perspective, it is unclear how these processes handle uncertainty surrounding the resulting visions and their implementation. This study reflects on previous visioning processes by systematically reviewing the scientific publications on participatory visioning in passenger transport. The review identifies possible improvements contributing to a systematic approach that produces concrete visions and actions to deal with uncertainties surrounding the vision and its implementation. We address these improvements by proposing a robust and generative visioning framework, which combines the generative approach in Appreciative Inquiry (Ai) and methods to handle uncertainty in the Dynamic Adaptive Planning (DAP). The framework is illustrated in a case study of the Southwest area of the Dutch city of the Hague that involved over 50 participants in a survey and two workshops. The process produced a vision for the mobility system of the area, a set of measures to realize it (i.e. pathways), and concrete actions to ensure that the pathways are robust against different futures that can affect the implementation. The approach can help planners, policymakers, and researchers in designing a visioning process that helps participants to better appreciate the temporal dimension of the visioning process and improves their awareness regarding the need to safeguard policy interventions against possible impacts of (un)certain future events.

## Introduction

The need for a more sustainable urban transport system is widely acknowledged but the transition toward it has been slow^1^. Visioning or a process to formulate a desirable future state in a participative manner is an essential tool in making a transformative shift towards sustainability^2^. Vision provides a guiding light towards a shared desirable future that stakeholders involved can progress toward and defines rationales they can refer to. Furthermore, visioning is a method of future inquiry that can support the structural and mental changes required for a sustainability transition^3^. It has been used to generate the strategies, policies, and actions necessary to achieve shared desirable goals^4,5^. In addition to these tangible outcomes, visioning has been observed to provide benefits that are desirable to the sustainable and transformative policy-making process, such as building capacity and knowledge, engaging community, mobilizing supporting, and inducing accountability from stakeholders^6,7^.

In transport planning practice, visioning has been well adopted and widely used as a method to support explorations of possible futures concerning urban environments and their associated transport system^8^. However, its applications are challenging for two main reasons. First, it can be difficult for stakeholders to identify and agree on what existing challenges and issues concerning the transport system should be addressed. This is due to the perceived quality of the urban transport system. Further, the importance placed on different parts of the system (e.g. road, footpath, or cycle path) can be different for each group of stakeholders (e.g. individuals, local businesses, local authorities) and sub-groups (e.g. cyclists and car drivers)^9^. With the diverse perceptions of what transport issues are, the priorities to solve them are likely to be different or even unclear as well. Particularly, in a context where the levels of transport services and quality of infrastructure are sufficiently high, such as in the Netherlands. Second, visioning processes in transport planning may not explicitly address different uncertainties associated with the long-term nature of visioning. The outcomes of these processes are often static visions that are set in a distant future with linear pathways toward them based on the current social, political, and technological trends^10^.

Uncertainties in transport policy and decision-making process are prevalent, particularly with trends such as digitalization, sharing economy, global pandemics, and climate change. These trends and their implications on political, economic, social, technical, environmentalal, and legislative aspects can alter future urban transport systems. They can influence visions, available options, their viabilities, and the likelihood of successful implementations of the associated options or pathways towards desirable visions. The uncertainties in decision-making processes are typically concerned with (1) the future state of the world, (2) how the state is considered, (3) policy and decision outcomes, and (4) the importance each stakeholder places on the outcomes^11^. Certainties on these four aspects are often unattainable as precise knowledge of all aspects is nearly impossible to obtain. The inadequacy of knowledge can range between complete certainty, a clear enough future (Level 1), alternate futures with probabilities (Level 2), a few plausible futures (Level 3), and Deep uncertainty in which there are many plausible futures (Level 4a) or unknown future (Level 4b)^12^.

Previous studies have recognized some of the above challenges and suggested possible measures to enhance the visioning process in transport planning. For example, the inclusion of wild cards to stimulate creativity^10^, citizen involvement through a mapping exercise^13^, the implementation of a quantitative multi-actor and multi-criteria analysis^14,15^, and the integration of the Delphi method and scenario planning into the visioning process^16^. However, these studies addressed uncertainties surrounding the visioning process and the implementation of the resulting visions at Levels 1 or 2 (i.e. a clear enough future that can be predicted with probabilities). Furthermore, these studies often approach the visioning process with a supposition that the issues and desirable conditions of the transport system can be readily identified and agreed upon by stakeholders. We argue that identification and reaching a consensus on urban issues can be challenging, because, in addition to the uncertainties mentioned, each stakeholder may also have a unique mental model or perspective and an individual set of priorities on the subject concerned^17^.

This research makes scientific contributions to the field of transport policy and decision-making process by highlighting gaps in the visioning process applied to transport cases, proposing a framework to resolve them, and illustrating its effectiveness in a case study. The systematic review is exploratory and focuses on (1) how related stakeholders and citizens were involved in the visioning process, (2) the methodologies utilized, particularly how they addressed the uncertainty surrounding the visions and their associated actions, and (3) the outcomes of the process (Sect. “Literature review: participatory visioning in transport”). The challenges identified in the review are then addressed with a proposed robust and generative visioning framework (Sect. “Robust and generative visioning framework”). The framework is then applied to a case study with real stakeholders in the Southwest area of Hague city, the Netherlands (Sect. “Application of the framework to the case study”). The outcomes of the process and their implications are then reflected upon and discussed before the research is concluded (Sect. “Discussion and conclusion”). The societal contributions of this study are the purported improvements to the visioning process that can support practitioners and researchers in formulating visions for a complex system, such as urban transport, in a robust and generative manner. In addition, this research is a product resulting from close collaboration and knowledge exchanges between the municipality mobility planning officers and the research team.

### Ethics approval

The Ethics Assessment Committee Faculty of Law and Nijmegen School of Management (EACLM) of Radboud University Nijmegen has reviewed the application of this publications, registered under EACLM number 2022.19. The committee declared that this research project is without ethical concerns and further ethical review is therefore not necessary. We confirm that the manuscript has been read and approved by all named authors and that there are no other persons who satisfied the criteria for authorship but are not listed. We further confirm that the order of authors listed in the manuscript has been approved by all of us.

## Literature review: participatory visioning in transport

We carried out a systematic search for scientific literature that documented how participatory vision making has been applied in the transport sector. The following search term: “Vision* and Transport OR Mobility and Sustainability and community OR participator* OR citizen OR stakeholder” was used in the Scopus database to identify 108 articles. The articles were screened by their abstracts to select only articles that report on a visioning process in the transport sector that are participatory; they should involve a group of participants, community, stakeholders, or citizens. Each article was reviewed by one of the authors. In case of doubt, multiple reviewers evaluated the article. After the abstract screening, 29 articles remained and one article was included on the recommendation of an expert. In total, 30 scientific articles were reviewed in detail and the full result of the review can be found in Online Appendix A. The studies reviewed primarily took place in Europe and the US, with a broad range of units of analysis that encompass a neighborhood, a city or multiple cities, a country, or a continent. The review results are discussed in this section, with a focus on the objectives of vision making processes, the methods used, how these studies handled uncertainty, and how the outcomes of these visioning processes were linked back to practice.

### Objectives and purposes

Generally, participatory visioning is implemented in these studies for five main reasons: (1) Visioning and pathways, (2) Strategies & decision-making support, (3) Policy assessment, (4) Elicit stakeholders’ preferences, and (5) Scenario building and evaluation. These objectives can overlap and are not mutually exclusive, see Table 1 for the five objectives and the references from the literature reviewed. Several studies explicitly mentioned sustainability or elements of it (e.g., livability or environmental friendly) as a normative value of their process (e.g.,^18–20^). Others address a more tangible characteristic of the transport system, such as compact city development^21^ or free of fossil fuels^22^. The nexuses between the transport system and other systems within the urban environment are also recognized and explicitly addressed by some of these studies, such as energy^23^ and consumption^19^.

**Table 1:** Five objectives of the participatory vision making process observed in the literature.

Objectives observed (# of studies)	References
**1. Visioning and pathways (5)-** to formulate a desirable future and define pathways toward achieving it	^19,21,24–26^
**2. Strategies & decision-making support (16)** – Use as a process to support the development of policies and strategies and decision-making processes	^13,15,16,20,22,23,27–37^
**3. Policy assessment (4)** – to evaluate planned or proposed transport development and implementation of sustainability solutions	^18,38–40^
**4. Elicit the preferences (2)** – to identify information on the perceptions and concerns	^41,42^
**5. Scenario building and evaluation (3)** – to develop and evaluate different future scenarios	^14,43,44^

### Process, participation, and methodology

The visioning processes of the reviewed studies generally consist of two main phases. The first phase is the divergence phase, in which participants are involved and express their ideas, preferences, and future desirability. The second is the convergence phase, in which information and data collected in the first phase are interpreted, analyzed, and processed, into a vision or a set of visions. Sometimes these visions also include potential actions to attain them, but often these actions are rather vague in terms of what needs to be done when it needs to be done, and who’s responsibility this is. The divergence and convergence phases are implemented through a variety of participatory methods with a diverse number of participants presented. The general means of engagement in these visioning processes are workshops (19 studies; e.g.^27^) and focus groups (3 studies; e.g.^19^). In certain studies, preparatory activities for these participations are included, such as literature reviews (three studies; e.g.^39^), desk research (four studies; e.g.^29^.), surveys (seven studies; e.g.^30^), and interviews (11 studies; e.g.^16^). In some studies, specific methodologies, such as the Delphi method (three studies; e.g.^26^.) or the Multi-actor Multi-Criteria Analysis (three studies; e.g.^15^.) were utilized. Auxiliary equipment, such as photographs (one study;^28^), and non-working prototypes (one study;^42^), was also utilized in some studies as supporting probs to support the process. The number of participants involved in the reviewed studies ranged from 12 participants in one workshop^36^ to 178 over a series of workshops^35^, however, several studies did not report the exact number of participants involved (9 studies; e.g.^33^.). Examples of the roles of participants represented in the visioning process were experts, researchers, citizens, and representatives of government or public agencies.

### Coping with uncertainty

The studies reviewed appear to have different approaches concerning uncertainty. Three different clusters are identified (See Table 2); (a) do not recognize or address uncertainty (21 studies), (b) recognize but do not explicitly deal with uncertainty (3 studies), and (c) recognize and addresses uncertainty (6 studies). The six studies that recognized and addressed uncertainty in their processes explicitly use two different methods. Namely, (1) scenarios building^14,34,43,44^ and (2) expert consultation^16^.

**Table 2:** Approaches concerning uncertainty in the literature and the methods employed to address it.

Uncertainty neither recognised nor addressed (21)	Uncertainty recognised but not addressed (3)	Uncertainty recognised and addressed (6)
^13,15,18–23,26,28,29,31–33,35–42^	^24,25,27^	*Scenarios building* ^14,30,34,43,44^*:*
		*Expert consultation* ^16^*:*

### Connection to practice

The outcome of the studies reviewed generally consisted of visions and, sometimes, associated actions to realize them. However, these outcomes differed widely in their forms (e.g., a set of criteria for desirable futures or policy recommendations, or objective indicators) and how these results were connected back into real-world policymaking and practices that instilled the processes in the first place. We classified the studies by how their results were connected with the real-world policymaking process, which ranged from no connection with practice (Isolation), to direct integration with policy practice, and everything in between. Table 3 provides an overview of the different categories which are not mutually exclusive. For example, the vision crafted by Borén et al.^22^ fed into the decision-making process through an initial development plan that was drafted based on it. Moreover, all local planning authorities involved in Cochrane et al.^32^’s process drew up a series of spatial strategies as fundamental planning frameworks.

**Table 3:** Four levels of connection to the practice.

Connection to the practice (# of studies)	References
**1. Isolated** (5)—results of the studies have no connection to policymaking, communication to the parties concerned is not reported or there is no direct link to the implementation or policy making process	^14,19,36,40,44^
**2. Informed** (7) – the results are communicated back to the participants but lacked a connection to the larger system to which the vision making relates. In a few cases, it is framed as a learning experience for participants and/or also validate the results by experts ^16^, or to observe the public reaction towards the proposed implementation	^16,20,25,31,41,42,45^
**3. Inputs and recommendation provision** (13)—outcomes of the process provide inputs for future research, practical exercise, or policy recommendations for the parties involved but with limited or no clear information on how these inputs and recommendations would be adopted or implemented	^13,15,18,23,26,28,29,33,34,38,39,41,43^
**4. Direct implications** (4) – clear and tangible links between the visioning process, its outcomes and policy practice	^22,27,30,32,37^

### Literature review findings

The results of the literature review above highlight three challenges concerning the visioning processes described in the papers. First, there is a lack of a systematic approach to visioning processes in these studies. The observed wide variations in participation formats, methods involved, and ways of reporting among the studies reviewed may be linked to the diverse range of objectives and contexts of these studies. However, a systematic and methodic approach with a guiding principle on how the process should be organized that enables adaptability to a context can help to improve the transparency, reproducibility, and credibility of these visioning processes is missing.

Second, most of the studies reviewed do not recognize or address uncertainty in their processes, while those that do are likely to assume that visions are static desirable points in the future with a set of associated actions to achieve them. The methods used to handle uncertainty in the reviewed visioning literature are expert consultation, scenario planning, and external influence analysis. These methods support the exploration of possible futures but do not help identify concrete actions to mitigate or capitalize on changing circumstances and to ensure that these visions can still be reached. Additionally, the methods are suitable only for uncertainties that have a sufficiently clear future, can be predicted with probabilities, or have few plausible alternatives (Uncertainty Levels 1–3). However, vision making is about a long-term future that is highly likely to encounter unforeseen changes (i.e. many plausible or even unknown futures – Uncertainty Level 4 or Deep uncertainty), such as the emergence of a new strain of the infectious disease, availability of self-driving vehicles, a social dystopia, or a global climate disaster. These plausible futures can affect both the realization of the vision, the measures to reach the vision, as well as the consequences of the vision. In the context of changing and unknown futures, a more flexible framework can be more favorable in place of a more static approach, such as those that backtracks from the imagined future^46^.

Third, we identified diversity in the level of explicitness in the resulting visions and a lack of clear connections to real-world policy and planning practices in the reviewed literature. Concerning the diverse levels of explicitness or concreteness in the resulting visions, some studies provided detailed desirable criteria of the future transport systems^14^ or indicator selection and target levels that are combined to form a vision^36^. Others report less detailed outputs that leave room for multiple interpretations (e.g.^23,31^.). Although diversity can come from the uniqueness within each study, a higher level of explicitness or concreteness in the resulting visions can be useful in the implementation stage or in linking the results back to the practice. It is also observed that the number of reports on the operationalization of the results of these vision making processes in these studies are limited. In several cases, it is unclear how vision making is linked back to practice in terms of which actions need to be taken at which moment in time and who is responsible to do so. Moreover, in general, the studies are rather vague about how the outcome of visioning are related to the transport planning process or to the initial motivation that triggered the visioning processes. Although researchers involved may not be able to directly influence how a visioning exercise connects to practice, particularly at the time of reporting, transparent reporting on this matter can be useful.

## Robust and generative visioning framework

**Figure 1:**
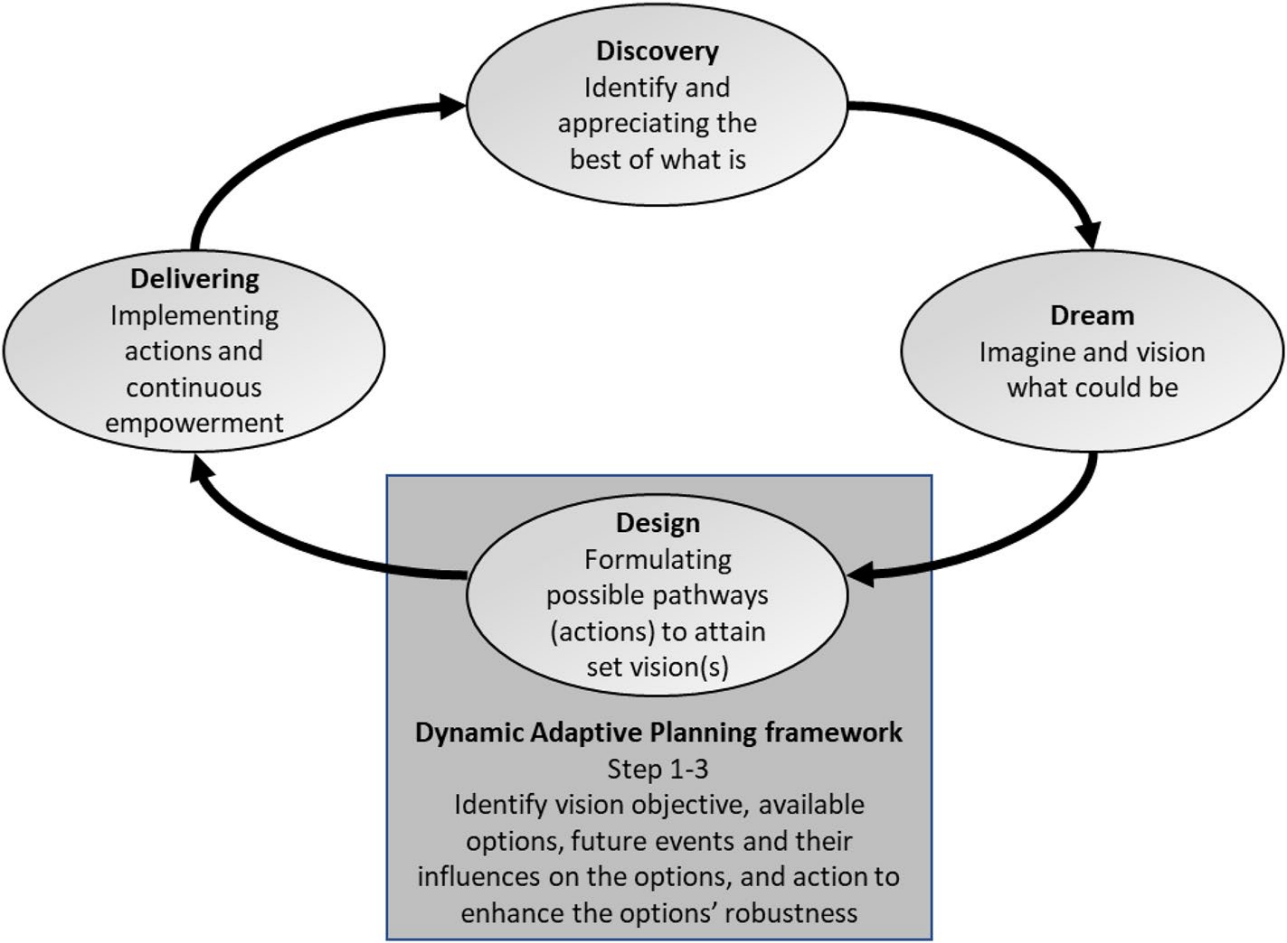
Robust and generative visioning framework, Adapted from^47^.

The findings from the literature have inspired the authors to develop a robust and generative visioning framework to address the three challenges found in the literature review. The proposed robust and generative visioning framework combines the Appreciative Inquiry (Ai) and the Dynamic Adaptive Planning (DAP) frameworks. It made up of four phases that are built on the Ai framework: Discovery, Dream, Design, and Delivering. Selected DAP steps are integrated with the Design stage of Ai. The proposed framework, as visualized in Fig. 1, provides a general approach for vision making in transport planning. The Design stage includes (i) the identification of possible future trends that can influence the pathways or sets of possible measures to realize the vision; and (ii) exploration of concrete actions to make them robust against these influences. Moreover, the methods used in the different phases can be adapted to the capabilities and wishes/needs of the stakeholders. For example, organized neighborhood walks or community interviews can be methods to engage stakeholders in the community in the Discovery and Dream phases.

By integrating Ai and DAP into one framework and translating this into a visioning process, the framework distinguishes itself from other visioning processes in three manners. First, the inclusion of Ai provides a generative approach that emphasizes identifying and strengthening existing positivity within the transport system that would lead to making the desired change^48^. The approach does not overlook existing problems but seeks to frame them in a positive light and is suitable for unclear system problems such as those in a contented situation.

Second, Ai also provides a coherent structure for different stages of the process (typically consisting of Discovery, Dream, Design, and Delivering) that enables participation from stakeholders and related communities with a variety of backgrounds and capacities^47^. The inclusion of the adaptive principles from the DAP framework yields the benefit of intrinsically encouraging participants to be explicit in their vision and actions required to actualize it. The requirement to be explicit by formulating concrete actions reduces the vagueness usually observed in studies generating visions and associated interventions. Moreover, a more concrete vision provides a better starting point for decision-makers to start a policy evaluation. As the integrated Ai and DAP framework includes several phases with different aims, it allows for the inclusion of a variety of stakeholders at different moments in the vision making process.

Third, the inclusion of DAP enables the process to handle different uncertainties that can influence the implementation of actions associated with visions (i.e. pathways)^49^. The framework helps to identify the objectives of the vision, available options (pathways), the underlying assumptions of a given plan, future events and their influences on the options, and action to enhance the options’ robustness. The framework enables the resulting pathways toward visions to be adaptive in any given futures (Uncertainty Level 4 – Deep uncertainty). The inclusion of DAP can be seen as an improvement to the visioning process in previous studies that included methods, such as Scenario Planning and Delphi technique, that can cope with uncertainty in a few plausible futures (Uncertainty Level 3).

## Application of the framework to the case study

**Figure 2:**
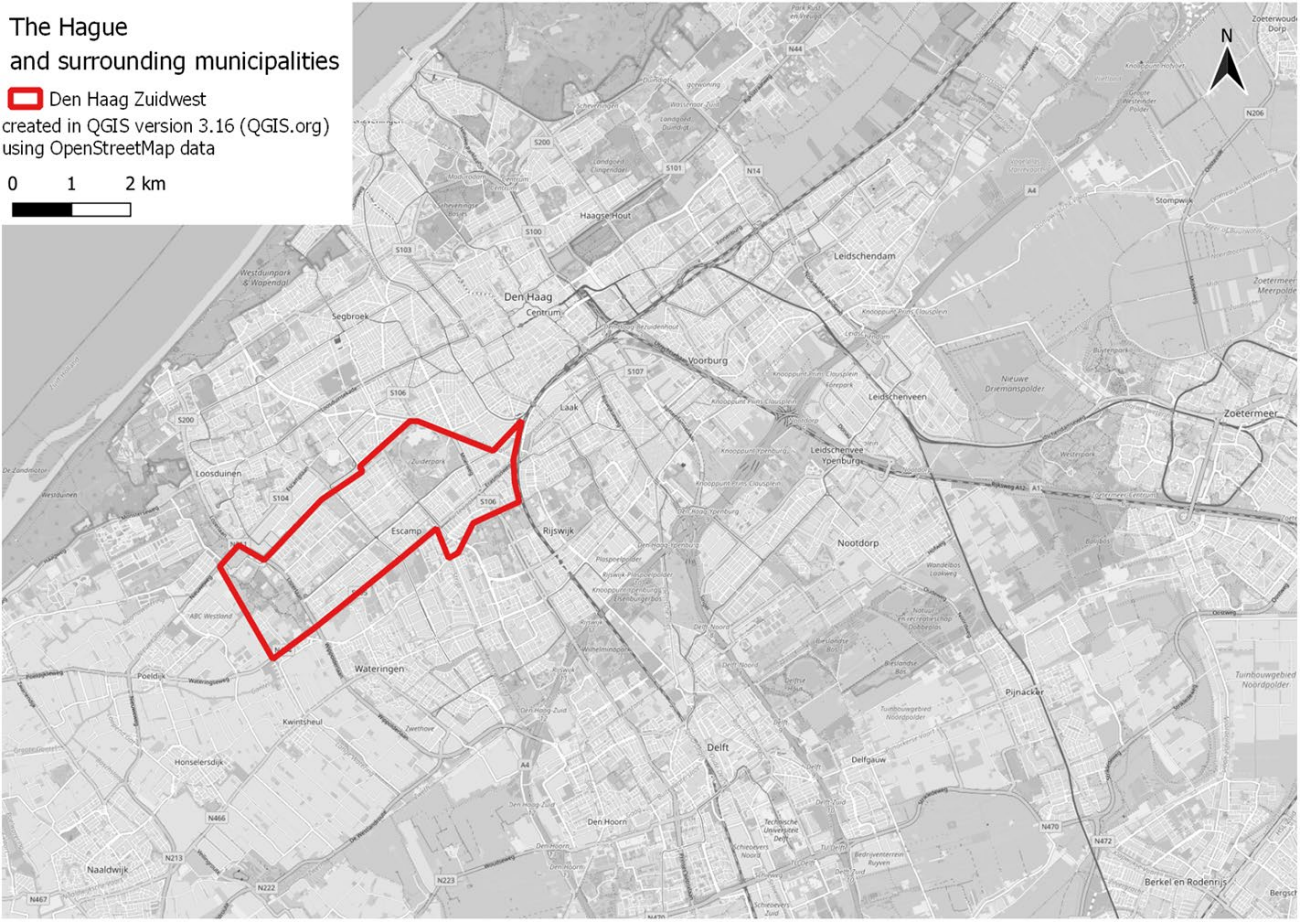
Area of interest: Southwest area of Hague city (Dutch: Zuidwest Den Haag).

In this section, we present the case study in which we applied the robust and generative visioning framework to illustrate its application process and the outcomes in practice. The case study is the southwest area of the Hague city. The urban area is located close to the city center (Fig. 2) and is part of the Escamp district, the largest and the most populated district of the city (approximately 130,000 residents in 2019). It used to be a vibrant residential community back in the 1950s but is currently faced with a range of urgent socioeconomic challenges that include poverty, polarization, low social cohesion, lack of health services, high unemployment, isolation, and domestic problems^50^. The municipality seeks to improve the area by planning infrastructure and residential developments in some neighborhoods within the districts, which will include the construction of housing, community facilities, parks, streets, and green areas. These developments are detailed in the Structure Vision for Southwest the Hague (Dutch:Structuurvisie Den Haag ZuidWest), which will be implemented in the next 20 years^51^.

The area is served by good quality public transport networks; a railway station, two tram lines, and five bus routes that combined provide public transport access to the local and surrounding areas. It also has a good road network that connects the area to the city and beyond and dedicated bicycle lanes and walking routes along most of the streets. However, the overall proportion of sustainable trips made by bicycle and public transport in the area is lower than the city average^52^. The net number of car trips generated here accounts for nearly 30% of all daily car trips in the city. Other challenges related to the transport system highlighted by previous and ongoing works (e.g. Mobility Atelier in 2019, Participation of the Mobility transition in 2020, and Structural Vision in 2021), include congestion on roads, limited parking, overdue renewal and maintenance of transport infrastructures, road safety and cyclists and pedestrians’ safety.

**Figure 3:**
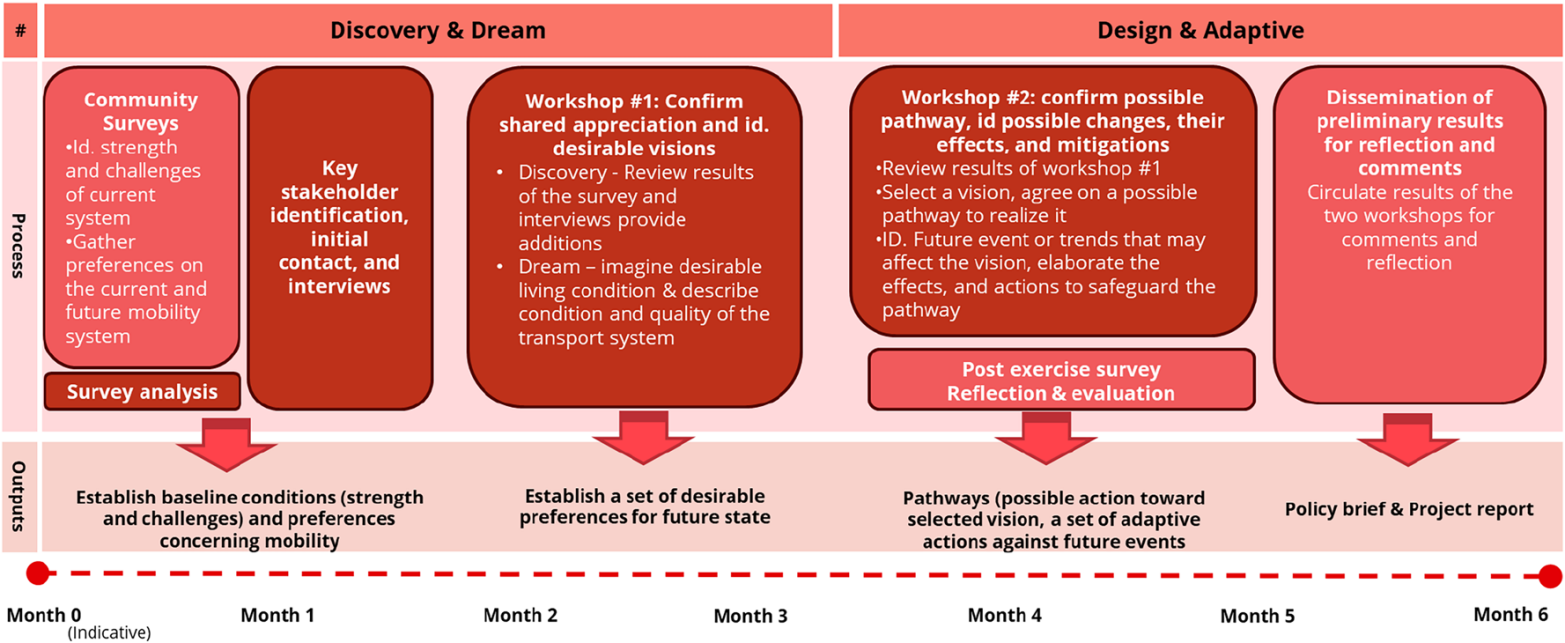
Process to implement a robust and generative visioning framework for the case study.

The case is suitable for the proposed visioning framework because the transport system of the area is currently adequate and no serious transport problems are observed, making it challenging for its stakeholders and residents to identify long-term desirable improvements. However, with the expected increase in housing units by 30% the area is faced with possible aggravation in congestion, air quality, and traffic accidents. The sustainability of the future transport system is a major concern of the municipality. In this project, we applied the first three phases of the robust and generative visioning framework, namely the Discovery, Dream, and Design phases. The delivering or implementing phase was not carried out as it requires a mobilization of resources that goes beyond the scope of the study. The three phases mentioned were carried out through two main research activities: a public survey and two workshops, which involved residents and key stakeholders from the area with different ages, backgrounds, and nationalities. The Discovery and Dream phases were implemented together in the surveys and the first workshop. The design and adaptive phases were addressed in the second workshop. The process is depicted in Fig. 3 and is described in detail below. The activities carried out with participants reported in this manuscript has been carried out in accordance with relevant guidelines and regulations. All the identities of the participants have been anonymized and informed consents were obtained from the participants. The need for ethical approval was waived by the Ethics Assessment Committee Faculty of Law and Nijmegen School of Management (EACLM).

### Community survey and initial key stakeholder interviews

After initial meetings between the research team and the municipality to co-develop the project objectives and process, the research team formulated and conducted a community survey. The survey aims to collect the baseline information: community opinions and their satisfaction with the transport services and infrastructures. Respondents were also asked about their expectations of future mobility systems, such as more space for walking and cycling and the provision of shared mobility services. The survey format was a mix between a travel survey and semi-structured interviews with stakeholders who lived in or traveled through the area to record additional opinions on future mobility and other aspects, such as their satisfaction and expectations.

The surveys were carried out in person on two occasions; in a marketplace (6th July) and in a location close to the community’s supermarket (8th July). In total, we interviewed 41 respondents, of which 26 were female, 10 were male, and 5 preferred not to state their sexes. The majority of the respondents lived in the area (86%) and had an average household size of 2.3 people. The results of the community survey highlight high proportions of multimodal travelers (95%) and satisfied respondents (90%). A summary and selected rationales behind their inputs to the survey are provided in Table 4.

**Table 4:** Summary of and selected outcomes from the public survey and interview (n = 41).

Daily mode of transport (% of respondents)	95% use more than one mode daily; walking (63%); cycling (59%); bus (34%); tram (41%); car (27%); scooters (7%); getting a lift (5%); shared vehicle (5%)
% of respondents satisfied or highly satisfied with…	The transport system overall (90%); walking (77%); cycling (81%); bus (76%); tram (80%); car (67%); scooters (50%)
Satisfaction with the current transport system	Walking and cycling are healthy; easy access to public transport stops; availability of cycle paths; South Park is beautiful; Good accessibility within the district; Availability of parking space and flexibility; Tram frequency is sufficient
Dissatisfaction with the current transport system	More bicycle paths and safe crossing; No separation between mopeds and cyclists; monotonous cycling routes; stolen bicycles, lack of security; Poor infrastructure e.g. sidewalks
% of respondents express a desire or highly desirable relationship with…	More space for walking, bicycle, public transport (90%); Sharing mobility (38%); Underground parking (25%); Personalized transport (34%); Electric vehicle (39%); Self-driving vehicle (8%)
Desirable Quality and Features of the future mobility system	Personal transportation with renewable/clean energy; small-scale public service (4 people.) with high frequency; a separation between scooters/bicycles; Road safety for all children; Slower traffic, narrower roads, and central parking with limited access for cars in the neighborhood; More greenery and play areas; accessibility for all, more stores and facilities in close proximity; affordable public transport for vulnerable groups (students, seniors), Less private car use
The rationale behind the stated desirable	EVs are the future; parking spaces are occupied by non-residents; there is a need for personal vehicle ownership; public transport needs more space and to be more affordable; better accessibility and frequency of public transport

### Stakeholder workshop #1: Shared appreciation and visioning

We shortlisted with the municipality nearly 30 stakeholders from diverse backgrounds who are related to the local transport system, such as residents’ representatives, local business associations, public transport providers, bicycle parking directors, policy advisors for special needs mobility, and the municipality planning officers. However, the Dutch’s Covid restriction at the time limited the workshop capacity to 20 persons including facilitators, so invitations were sent to 14 individuals within the shortlist. Care was taken to ensure a diverse representation of different groups. These key stakeholders were contacted by email to invite them to join a two-session workshop on mobility in the Hague Southwest (Dutch: Bewegen in Den Haag Zuidwest). A short interview with each interested participant was conducted by telephone. Five stakeholders were reached. The duration of the interviews is 15–20 min on average and includes questions on their role in the area’s mobility system, their positive experiences with the system, and their expectations for the task force workshop. The interviewees were a resident of the area, a strategic advisor for a public transport provider, a cycling instructor, a bicycle parking director, and a policy advisor for special needs mobility. The results of the public survey and the phone interviews were analyzed and provided as inputs into the first stakeholder workshop.

The first workshop was organized at a local theater in the area on 15th September 2021. The workshop lasted approximately two hours and eight participants attended the meeting with the following expertise: cycle advocate, planning officer (municipality), special need mobility group, environment, housing, citizen, public transport provider, and housing organization. The session began with a brief welcome from the organizers and an icebreaker session. A representative from the municipality presented its sustainable objectives and future challenges of the area to set the scene. The workshop’s two main activities, Discovery and Dream, were then introduced. In the Discovery session (which lasted 40 min), the results from the public interview were briefly presented. The participants were then divided into two groups of mixed backgrounds and asked to review the results and add the charecteristics and qualities of the area they appreciated the most. A facilitator was available in each group to support the discussion systematically and three research staffs were present to support the session in general. In the Dream session (which lasted 40 min), the current situation of the area transport system and its possible future were presented. The respondents were then asked to consider the ideal living conditions for themselves and people in the area in 20 years (the year 2041), with a particular focus on transport. After this activity, the participants returned to the plenary for a brief reflection on the session and the first workshop was then concluded.

**Figure 4:**
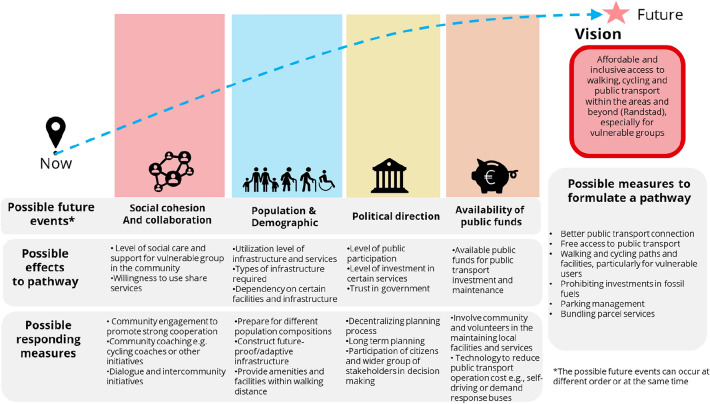
A summarised outcomes of the stakeholder workshops.

Data collected during the first workshop consisted of memos, audio recordings of each group, notes of participants, and reflections from researchers and municipal employees present at the workshop. The audio recordings were transcribed and the contributions of the participants and reflections of the facilitators were analyzed and clustered into themes. These themes include transport, green areas, built environment, and social and well-being. For example, in terms of transport, participants believe the accessibility between the area and its surroundings is good but it is desirable to enhance the inclusion of vulnerable groups and public transport accessibility in particular. Finally, the themes were summarized as tentative visions for future mobility in DHZW (see Online Appendix B). The respondents selected one of the visions for the next activity—*Affordable and inclusive access to walking, cycling, and public transport within the areas and beyond (to the Randstad area), particularly for the vulnerable groups (Fig. *4*, Vision)*. We compared the final results with previous studies on future mobility (e.g., the Hague Participation Process for the Mobility Transition (2019–2020) and the Structural Vision for the Southwest area), which shows that they are comparable in elements such as inclusiveness, accessibility, and promotion of nonprivate vehicles (see Online Appendix B). The research team also extracted a set of pathways, such as better public transport connections, free access to public transport, and parking management from a list suggested by the participants during the first workshop (Fig. 4, possible measures to formulate a pathway).

### Stakeholder workshop #2: Pathway exploration and design

The second workshop took place on 14th October 2021 at the same location with eight participants (of which five participants were present in the first workshop) with similar expertise to the first workshop. The results of the first workshop, in the form of a shared vision and possible measures to formulate a path achieve the vision, were presented to the participants for their comments. The research team then asked the participants to adopt the vision and the pathways shown in Fig. 4 as a starting point for the vision of the area. The participants were divided into two groups, each with a facilitator to help them identify possible futures and trends that can affect the tentative pathway. Four key external changes/future events can be identified from the possible futures and trends defined by the participants: social cohesion and collaboration, population and demographics, political directions, and available public funds. Based on this categorization, we clustered the possible external effects which can change the pathways and the measures to formulate the pathway (Fig. 4, possible future events). We then asked the participants to identify how these future developments and trends might influence the pathways (Fig. 4, possible effects on the pathway). For example, a decrease in social cohesion can reduce social care in the community and support for vulnerable groups, as well as the willingness to participate in shared mobility services. The next step in the process was to identify possible actions and measures to safeguard the pathway from not leading to the desired vision or taking advantage of the changes occurring (Fig. 4, possible responding measures). After these steps in the two different groups, the participants convened back in the plenary to review and reflect on their works and the process, such as the challenges in considering future events, to conclude the workshop. The data collected during the workshop was analyzed in the same manner as at the first workshop.

### Outreach and dissemination

After the two workshops, we compiled the results of the process into a report for the municipality and the participants of the workshops. A preliminary report was first composed and circulated among the participants for general comments on the process and its outcomes to provide them with an opportunity to provide additions. A brief survey to evaluate the process was also sent to the participants, but due to a limited reply, the results are not included here. After the report was finalized in May 2022, the municipality circulated the report within its organization and organized an internal event to disseminate the results of the process on 9th June, 2022. Officers involved in the planning of the mobility of the municipality and in the process to formulate the Structure vision for the area were presented and received copies of the report.

## Discussion and conclusion

Vision making has been widely adopted and implemented in transport planning and policy making. In this research, we reviewed the literature to identify possible improvements to the vision making processes in the transport domain. Three possible improvements were identified, namely (1) a systematic approach to visioning processes, (2) explicitly addressing (Deep) uncertainty in which there are many plausible or even unknown futures, and (3) a higher level of explicitness or concreteness in the resulting visions. We addressed these improvements in the form of an integrated robust and generative visioning framework. The framework distinguishes itself from other visioning approaches by its focus on the following aspects:

1. Generative and discursive: the process revealed positive experiences and qualities of the transport system appreciated by the participants. The focus on personal experience also results in a more relatable vision for participants. Moreover, the process encourages participants to explicitly identify possible measures or pathways to actualizing the vision concretely either by themselves or by other stakeholders. This process can help to empower stakeholders and stimulate discussion among them.
2. Systematic and explicit process: the integrated framework provides a step-by-step approach to the visioning process which encourages participants to be explicit in their vision and the actions required to actualize it. Moreover, since the process consists of different stages it allows for the inclusion of a diverse group of participants and stakeholders at different phases in the process with diverse methods fitting the background and capability of the participants.
3. Future-proof: the concrete actions, possible future events, and their impacts on the actions enabled participants to address uncertainty surrounding the pathways toward their vision. Hereby, the adaptive process supports participants to define actions to prevent negative impacts on the pathways toward visions and the resulting pathways are thus adaptive and robust in any given futures.

The framework was applied to a case study in the Southwest area of the Hague with a group of local stakeholders. The application illustrates how the framework can help to formulate visions of the future mobility system generatively and assist in addressing a variety of future uncertainties concerning its implementation. This work has several implications for theory. The literature review clearly showed the gaps in the existing visioning approaches and thereby opens the way for systematic evaluation of participatory visioning processes. We also sought to resolve the gaps identified by purporting the robust and generative visioning framework and provided a first step in illustrating the application of the framework. And finally, the transition of the mobility system towards sustainability requires change at all levels, including or potentially even especially from citizens. Therefore, it is of high importance to include them in visioning processes. In practice, this structured explanation of the approach allows replication in various case studies. It facilitates the inclusion of citizens, the local community, or general users of the system in the visioning process.

Reflecting on the implementation of the framework, we highlight several lessons learned and the limitations of this study. First, we found the semi-structured interviews in the survey was a useful activity to engage with the public prior to the workshop sessions. Asking open questions on participants' desirable future daily travel and their preferences for a future transport system in the next 20 years, led to meaningful discussions that revealed participants’ lifestyles, preferences, and assumptions. It provided a starting point for the workshop sessions later on. However, we found that mentioning tentative changes in the local area (i.e. a plan to increase housing units in the area), can also tricker curiosity that steers the survey to focus on the tentative changes. In future work, care should be taken in the selection of a tentative change and how and when to present it to the public.

Second, the generative approach instills constructive interventions and enabled the participants to deal with the uncertainty involved in the visioning process. The generative approach enabled the researchers and facilitators to frame the activities with the participants in a way such that the focus was on the positive qualities of the local transport system. Moreover, the generative framework stimulated the participants to think positively while acknowledging existing challenges. Although critical statements and discussions on housing issues and space management were held during the survey and the workshops, the atmosphere, in general, was pleasant and cooperative. The participants’ feedback on the workshops revealed that they were generally optimistic about the methodology; the positive-focused approach enabled them to be creative and to have constructive discussions. Further research is needed to quantify and confirm this. Additionally, the adaptive framework enabled the participants to appreciate the temporal dimension of the visioning process and the need to safeguard the vision against possible impacts from certain future events. The process also resulted in concrete actions to enhance the robustness of the vision. However, we see a potential to develop the framework further by developing additional steps will be required to assign actions to actors in the process and moments in time and to design a monitoring process to ensure that the resulting vision is more futureproof.

Third, we found engaging participants in identifying desirable futures challenging. During the public survey and the workshops, we encountered similar challenges in visioning processes that were also reported by previous work – specifically on how to identify desirable futures. Additionally, we also found it challenging for the participants to perceive the implications of a given future threat (in this case, a significant increase in the number of inhabitants in the area). These challenges may result in contentment that hinders the visioning process (e.g., ‘there is no transport problem here” or ‘everything is already good enough’). Also, it may limit the creativity of participants and make them likely to suggest incremental changes to the existing (e.g., ‘perhaps more parking and more bicycle lanes’) rather than disruptive or out-of-the-box possibilities. We believe the additional time for group work and the inclusion of methods to support vision probing can be useful to facilitate out-of-the-box thinking, such as the use of a wildcard^10^ or a considering the needs of the future generation^53^.

Fourth, the capacity and capabilities of participants to engage in a visioning process should be considered to ensure broad participation. The proposed framework was successful in engaging with a wide range of stakeholders and citizen representatives from different backgrounds and ages because of the different phases and the possibility to include a variety of participation forms. However, the attendants of the focus group meetings were primarily middleclass or elderly Dutch nationals, with a sufficient level of legibility and who have the personal capacity (time & resources) to spend their valuable weekday evenings with the research and facilitation team. Representatives from other age groups and ethical minorities that reside in the area were interviewed in the survey but were not all present at the workshops. Another important aspect to consider is the time investment of the participants and their commitment to participate. Consideration of how different groups can be involved at different stages makes the process more participative and allows for the inclusion of diverse perspectives. Also, we found that close collaboration with the local authority in planning, designing, and excusing the process was the impetus to a successful visioning process.

Fifth, the number of participants in the process was sufficient to illustrate how the proof-of-concept framework can be implemented. For future practical cases, additional participants would be desirable. The limited participants in this study were due to resource limitations, the Covid-19 restrictions at the time, and the participation fatigue reported by the invited participants (several participation exercises took place in the area at the same time). To scale up the proposed visioning process, the involvement of citizens and local representatives in the Discovery phase is supposed to be fruitful. At this point collaboration with existing activities in the area can be set up. For example, participative initiatives such as local appreciation walks can engage citizens in assimilating information from each other. Additionally, organizing workshops online or in a hybrid format can broaden the reach. Smaller group meetings or workshops should be considered as they provide more opportunities to interact. Finally, the inclusions of group reflection and evaluations at various stages were helpful to take stock of and guide the process.

Future studies can consider the robust and generative visioning framework and how it is further applied based on the results reported here. Several improvements to develop the framework further were mentioned before, our first step will be to formulate additional steps (using DAP) to make the result of the visioning process more concrete and tangible. In the final workshop, stakeholders will assign the resulting actions from the workshop to themselves or other stakeholders involved. Moreover, they formulate a monitoring process to enable the visioning framework to cope with Deep uncertainty. Applications of the framework to other cases in the transport sector or different domains that are faced with similar challenges (e.g., energy or urban design) may also highlight possible enhancement. Additionally, a longitudinal study that provides a follows up on this case study (or others) over an extended period to examine how the results of participatory visioning are taken further by responsible stakeholders can be valuable. For instance, understanding how visions are adopted and implemented or result in policies that shift the current paradigm can help to improve the vision formulation process. Such studies are rarely reported in the scientific literature. Additionally, as the visioning process reported here does not include the implementation (Delivering) phase, processes such as decision-making or trade-off analysis between options are not addressed. As a direction for future research, we propose the inclusion of frameworks or methodologies that help to quantify the impact of pathways and their contributions towards sustainability goals (i.e. emission reduction and social well-being – see for example^54^). It is also notable that the vision considered in this study does not address environmental aspects of sustainable transport explicitly. Future studies can emphasize this aspect more strongly by framing the environmental challenges in the initial briefing with participants.

## Supplementary Information

Supplementary Information 1.

Supplementary Information 2.

## Data Availability

The datasets generated and/or analysed during the current study are not publicly available due to privacy of the particinpants involved but are available from the corresponding author on reasonable request.
