# Supplementary material for: Visioning future transport systems with an integrated robust and generative framework
Source: Sci Rep. 2023 Mar 15;13:4316. doi: 10.1038/s41598-023-30818-2 (PMC10016152; doi:10.1038/s41598-023-30818-2)
Supplement: Supplementary file 1 — Supplementary Information 1. [file 41598_2023_30818_MOESM1_ESM.docx]

**Appendix A: Literature reviewed in this study**

| Reference / context | Objective | Methods | Participants | Uncertainty | Outcome |
| --- | --- | --- | --- | --- | --- |
| [13] Losangeles country, California | Formulation of a development plan, collection and spread of information, gathering of citizen support | Workshop, mapping exercise, survey, marketing material collection | 150 and 50 participants from westside cities | Not recognized, nor addressed | Policy recommendations, key policy options, vision, strategies |
| [14] Europe | Evaluation of future scenarios | Scenario analysis, Multi-Actor Multi-Criteria Analysis (MAMCA), workshop, survey | 38 stakeholders in 5 groups | Yes, through scenario planning (finding a plausible future on which the vision is set) | Vision Map (desirable criteria of future transport system) stayed within the expert group |
| [15] Bristol and Newcastle, UK | Design of a new city logistics scheme | Workshop, Multi-Actor Multi-Criteria Analysis (MAMCA) | Logistics operators, supply chain managers, retailers, customers, citizens | Not recognized, nor addressed | A co-designed framework that can support policy-makers to design and implement new policies during the transitory period |
| [16] maritime ports around the world | Definition of plausible development paths of maritime ports | Expert interviews, scenario development, and analysis, online Delphi survey, expert interviews | 78 qualified experts | Yes, the reason why expert opinions are collected as the most reliable source for future predictions | Scenarios are used as a basis for development paths which are validated by experts |
| [18] Rural and Urban areas of Switzerland | Evaluation of solutions and their implications for a connected and sustainable lifestyle | Essay writing about personal visions, follow-up workshops, qualitative content analysis of essays | 105 pupils (10-12 years old) | Not recognized, nor addressed | Vision, Transition pathways, possible implications associated, possible risks and governance management, and concerns of participants |
| [19] New Zealand | Creating a vision as a basis for a participatory backcasting exercise | Backcasting, focus group, group discussion | 36 first-year university students | Not recognized, nor addressed | A set of normative goals as a Blueprint toward sustainable consumptions concerning housing, clothing, travel, leisure, and food. |
| [20] US suburban communities | Creation of guidelines for a more liveable community | One-on-one stakeholder engagement, dialogue at public meetings | Representative advisory committee, community members | Not recognized, nor addressed | Vision and design guidelines for the community; community dialogue |
| [22] Southeast Sweden | Exploration of a roadmap for a fossil-free transport system | Desk research, workshops | 30 experts from regional transport systems in southeast Sweden | Not recognized, nor addressed | Vision feeds into the decision-making process, public review, and initial development plan |
| [23] Germany | Transformation of the existing mobility and energy practices | Interviews, collection of documents, participant observations | Not reported | Not recognized, nor addressed | Assumptions and blind spots from an imaginaries perspective to help raise awareness and be more explicit in their assumptions and debate |
| [25] Germany | Creating a vision as a basis for a participatory backcasting exercise | Backcasting, Delphi survey, semi-structured interviews, workshops | 140 experts | Recognized but not addressed | Communication of the vision among the group (using workshop) but not broader |
| [26] Andalusia, Spain | Creating a vision as a basis for a participatory backcasting exercise | Delphi survey, semi-structured interviews | 40 stakeholders in two groups, normative actor the public, practitioners and decision-makers, academics | Not recognized, nor addressed | Using Backcasting process to determine actions required to reach the vision |
| [27] North Rhine-Westphalia, Germany | Analysis of the development of a sustainability strategy | Desk research, workshops | Experts, advisory board, inter-ministerial working group general stakeholder | Recognized but not addressed | Public consultation; parts of the outputs are adopted by the practice |
| [28] Singapore and Joho, Malaysia | Improving transit space management | Semi-structured interviews, photographic survey | 36 participants | Not recognized, nor addressed | Identification of issues for future studies and policy formulation |
| [29] a fictive case | Part of a process to support strategic decision making | Desk research, workshops | Researchers | Not recognized, nor addressed | Identified policies and actions are used as input for simulation scenarios |
| [30] Blekinge region, Sweden | Support the preparation of a regional planning process by broadening perspectives of the future, including uncertainty, and inspiring actions | Framework for Strategic Sustainable Development (FSSD), workshop, stakeholder survey, evaluation interview | 43 participants from 13 stakeholder organizations | Static - identification of possible key external factors and drivers that would influence the area of interest i.e. population and sustainability | Workshop summary report and Scenario development, Scenarios inform policy |
| [31] Birmingham and Bristol, UK | Definition of a civil engineer's brief | Clustering of aspirations about the city, workshops | 64 stakeholders | Not recognized, nor addressed | A learning experience for participants |
| [32] Urban areas, UK | Formulation of a plan to integrate housing and economic development; reflection on past development | Detailed documentary analysis and in-depth interviews | Officers and elected members in a range of local authority roles, members of community organizations, representatives of private sector bodies | Not recognized, nor addressed | Connection to planning and implementation of strategies defined: All authorities in the case study area were required to establish their initial Core Strategies, revising them over time. |
| [33] EU cities | Integration and evaluation of three projects to create a road map for bus system development | Assessment of project performance, modeling and surveys, and transferability assessments | Not reported | Not recognized, nor addressed | A vision and road map on how to develop the bus system of the future; key priorities for future developments |
| [34] Urban areas of the Netherlands | Generation of integrated options for strategic, long-term urban development policies | Backcasting, workshops | 100 experts (scientists and practitioners) | Yes, using scenario design based on two axes (population, collective/individual choice) | Visions and an overview of actions and necessary efforts and placed on a timeline starting from now until 2040 |
| [35] Six coastal cities in South Brazil | Establishing new groundwork to fulfilling alternative visions for the regional social-ecological system transformation | Workshops | 19 workshops, 178 participants | Not recognized, nor addressed | Perceptions of Ecosystem from the socioecological system perspectives; shaping political actions in an eco-system based management |
| [36] Sky Harbor neighborhood of central Phoenix, Arizona | Formulation of targets and indicators for neighborhood transportation access | Sustainability Solution Space (SSP), workshops | 12 representatives of the neighborhood | Not recognized, nor addressed | Indicator selection and the target levels that combined to form vision |
| [37] Mediterranean cities, Europe | Examination of barriers and opportunities of the implementation of RTEG | Discussion seminars with an interdisciplinary group of experts from different sectors, survey | 15 interdisciplinary experts from different sectors | Not recognized, nor addressed | Inputs and recommendations for the practice |
| [38] Doha, Qatar | Exploration of the impact of a planned transport development | Literature review, data collection, and site analysis focus group interviews, participatory observations, survey | 40 participants from governmental agencies, 40 end users, and citizens | Not recognized, nor addressed | Set of recommendations for ongoing policies or plans |
| [39] no specific region | Assessment of urban logistics pooling through a common definition of scope and indicators | Systemic literature review, group decision-making method based on consensus research, Multi-Actor Multi-Criteria Analysis (MAMCA) | 20 experts | Not recognized, nor addressed | Dashboard of indicators, database |
| [40] European and UK cities | Elicit information about citizens' perceptions and concerns, provision of information | Integrated Sustainable Assessment Framework, workshops, interviews | 44 experts and 14+15 citizens (separately) | Not recognized, nor addressed | Experts and layperson vision - possible measures toward the stated vision, induce social learning among participants |
| [41] Milton Keynes, UK | Elicit information about citizens' perceptions and concerns | Mock-ups and non-functional prototypes of the product and illustration to initiate dialogue in the citizen engagement workshops | Participants from a variety of backgrounds | Not recognized, nor addressed | Observation of public reaction toward the vision of how the MotionMap will be implemented |
| [42] Europe | Scenario building | Backcasting, workshops, scenario building and modeling, literature review | 30-40 participants per workshop including stakeholders from different social spheres | Yes, by simulating different scenarios | Building scenarios from visions and simulating scenarios |
| [43] Different regions within the EU | Scenario building | Environmentally Extended Multi-Regional Input-Output analysis to model scenarios, backcasting, simulation of scenarios, workshops | 30-40 participants from different regions | Yes, included in the model simulation | 19 scenarios of sufficiency (net reductions) and 17 of green consumption (shift in consumption patterns), no explicit communication or policy link |
| [44] Turin, Italy | Creating a vision as a basis for a participatory backcasting exercise | Interviews, workshops, focus group | Researchers, local experts, general stakeholders, and citizens | Recognized but not addressed | Vision and pathway communicated back to participants in a workshop |
| [54] Sacramento region, USA | Prioritization of ‘compact development’ (enable residents to use walk, bike, and public transport) as a part of smart growth | Small group workshops, large regional meeting | First small but diverse groups of citizens, then an event with 1400 business leaders, elected officials, and citizen planners | Not recognized, nor addressed | A preferred plan approved by officials and the basis for an official transportation plan |
