# Supplementary material for: Visioning future transport systems with an integrated robust and generative framework
Source: Sci Rep. 2023 Mar 15;13:4316. doi: 10.1038/s41598-023-30818-2 (PMC10016152; doi:10.1038/s41598-023-30818-2)
Supplement: Supplementary file 2 — Supplementary Information 2. [file 41598_2023_30818_MOESM2_ESM.docx]

**Appendix B: Vision of the ZWDH mobility system and a comparison with other studies**

Three main elements were identified as the key vision for the ZWDH mobility system (See the full vision, classified into four categories in Appendix B):

1. Affordable and inclusive access to walking, cycling, and public transport within the areas and beyond (to the Randstad area), particularly for the vulnerable groups
2. Availability of innovative mobility services and concepts (e.g., electric, shared, and micro-mobility)
3. Affordable public transport and fewer private car use and ownership

| **Future desirable from this vision exercise (2022)** | **Participation of the Mobility transition 2019-2020** | | **Structure Vision 2021 (mobility ambitions)** |
| --- | --- | --- | --- |
|  | **The Hague** | **Escamp district** |  |
| Affordable and inclusive access to walking, cycling, and public transport within the areas and beyond (to the Randstad area), particularly for the vulnerable groups | - Road safety and more room for vulnerable road users are highly desirable; Mobility should be inclusive  - Bicycle is the most affordable means to transport but more parking spaces are needed in the city and the neighborhoods-  - Affordable, speed and door-to-door connection are crucial criteria for public transport service | - Strong preference toward inclusive transport solutions than advanced and high-tech solution  - Better public transport access; with a strong neighborhood connection is preferred; fast tram connection  - More space and facilities for cyclists and pedestrians; active promotion of cycling  - Affordability is preferred to personalize | - Strengthening accessibility by public transport to the region, the Randstad, and beyond  - Improving and strengthening pedestrian connections, including to public transport,  - Improve bicycle connections within and to other surrounding areas  - No information on affordability |
| - Availability of innovative mobility services and concepts (e.g., electric, shared, and micro-mobility) | - A lack of awareness on shared mobility but an interest if they are affordable, easily accessible, and readily available  - Positive opinion on mobility solutions that contributes to a more livable city, e.g. subway, mobility hub, and light rail. | - A lack of keenness to use shared mobility due to inconvenient and also concern about using MaaS due to a potential lack of control and privacy issues;  - Still, access-based transport service has potentials;  - e-car charging facility should be easy access; | - Improving traveler choice and combination options (shared mobility and mobility hubs)  - Stimulate a transition to clean, space-efficient, and safe mobility  - Explore burden-mile delivery hub, sustainable transition hub |
| - Fewer private car use and ownership | - Car ownership remains important for its convenience but second-car will not be necessary if there is a competitive alternative | - High car ownership and usage;  - Parking facility and parking management are highly concerned issues  - Good access for cars remains essential if public transport access is not improved;  - Car remains the most convenient mode but 2^nd^ car should be discouraged | - Limited car traffic to several well-integrated urban and regional main road networks, promote car-free residential or low-speed streets (30 km/h) |
